# Pain management in inflammatory bowel disease: feasibility of an online therapist-supported CBT-based self-management intervention

**DOI:** 10.1186/s40814-021-00829-9

**Published:** 2021-04-16

**Authors:** Louise Sweeney, Rona Moss-Morris, Wladyslawa Czuber-Dochan, Christine Norton

**Affiliations:** 1 Health Psychology Section, King’s College London, London, UK; 2 Florence Nightingale Faculty of Nursing, Midwifery & Palliative Care, King’s College London, London, UK

**Keywords:** Inflammatory bowel disease, Chronic pain, Cognitive behavioural therapy, Self-management

## Abstract

**Background:**

Chronic pain is a poorly managed symptom of inflammatory bowel disease (IBD). Cognitive behavioural therapy (CBT) has an evidence base in functional gastrointestinal conditions and chronic pain. This study aimed to test the feasibility and acceptability of a 9-week online facilitator-supported CBT intervention, tailored for people with chronic IBD-related pain.

**Design:**

A single-arm pre-post design with nested qualitative interviews was used. Twenty individuals with IBD and chronic pain were recruited through an online IBD charity and had consented to research in a previous survey or responded to an online charity advert. Individuals who indicated a pain-interference score of ≥ 4/10 (Brief Pain Inventory) and met inclusion criteria were invited to take part. Outcomes included recruitment and retention rates, pain interference and severity, quality of life (QoL) and psychosocial measures.

**Results:**

Of 145 individuals contacted, 55 (37.9%) responded. Two individuals were recruited from the study advertisement. Twenty out of 57 (35.1%) met screening and eligibility criteria. Eighty-five percent of the sample engaged with intervention sessions and 55% completed at least 5/9 sessions. Eighty percent of recruited participants completed the post-intervention questionnaire at week 9. The mean score for overall acceptability was 43.4 (0–70). Qualitative feedback demonstrated the value of thought monitoring and facilitator support. Scores improved for QoL and pain self-efficacy and reduced for depression, anxiety, pain catastrophising and avoidance resting behaviour.

**Conclusions:**

Online CBT for chronic IBD-related pain appears feasible and acceptable. The study suggests positive effects for improving QoL and reducing psychological distress; however, online and face-to-face recruitment methods are recommended and establishing efficacy through larger randomised controlled trials is required.

**Supplementary Information:**

The online version contains supplementary material available at 10.1186/s40814-021-00829-9.

## Introduction

Abdominal pain is a key symptom of inflammatory bowel disease (IBD). Pain is a hallmark symptom for assessing disease activity, guiding treatment decision-making and serving as a key outcome in IBD clinical trials [1]. Yet chronic pain is a poorly understood and managed symptom of IBD. While 70–80% experience abdominal pain during active disease, up to half of patients continue to experience pain when disease is seemingly controlled as indicated by endoscopic and clinical disease markers [2]. Many patients also experience extra-intestinal joint or musculoskeletal pain [3]. Contributing factors to the aetiology of chronic pain include visceral hypersensitivity and dysregulation of the central nervous system (CNS) [2]. Recent research has shown that a range of psychosocial factors, including depression, anxiety and fear avoidance, are associated with pain [4]. Depressive symptoms and pain catastrophising have been shown to mediate the relationship between pain and pain-related disability in IBD [5]. This suggests that altering cognitive and behavioural responses and negative mood in relation to pain may have beneficial effects on reducing its severity and impact.

Treatment approaches to modifying pain in IBD have included pharmacological, psychological and dietary techniques, summarised in a review by Norton et al. [6]. There is a lack of consensus or guidelines on how to optimally manage long-term pain in patients with IBD. In many cases, the first-line treatment of chronic pain in IBD is pharmacological, but many of these approaches either risk gastrointestinal complications or have not been tested specifically in IBD-pain populations in large randomised controlled trials (RCTs) [7]. Treatment strategies used for chronic pain do not always relieve symptoms for patients with IBD [8]. Developing an individual’s coping, self-efficacy and ability to self-manage can lead to improvements in both mental and physical outcomes [9]. A growing consensus advocates the use of psychological therapies in IBD [10] with the aim of attenuating both psychological distress and associated symptoms.

Cognitive behavioural therapy (CBT) is one of the most widely used psychotherapies for chronic pain [11] and is a recommended treatment approach for psychological co-morbidity associated with long-term conditions [12]. It is based on the principles that low mood, unhelpful thoughts and behaviours are interrelated and can perpetuate physical symptoms [13]. Meta-analyses have demonstrated equal efficacy between different modes of CBT including comparable effects from face-to-face and guided internet-delivered CBT for psychiatric and somatic disorders [14] and adults with depression [15]. In a recent large RCT, both online and telephone therapist-supported CBT led to significant improvements in quality of life (QoL) and reductions in irritable bowel syndrome (IBS) symptom severity and impact [16].

In IBD, evidence supporting CBT is less conclusive. Mikocka-Walus et al.’s [17] RCT compared online or face-to-face CBT with standard care and found no changes in disease activity, QoL or psychological distress. However, sub-analyses demonstrated improvements in QoL at 6-month in individuals with greater baseline distress. Jordan and colleagues [18] screened for low mood and moderate anxiety and found that face-to-face CBT led to improvements in QoL and reductions in psychological distress and symptomatic disease activity. Therefore, despite equivocal evidence of CBT in IBD to date, further work is needed to explore its efficacy in appropriate key sub-populations of IBD. This includes individuals with psychological comorbidity but also chronic IBD symptoms which have a psychological component, such as fatigue [19] and pain.

For chronic pain associated with a range of conditions, CBT has shown to have a positive effect on mood and pain-related impact [20]. These include tailored CBT interventions which are formulated specifically for pain but may have downstream effects on secondary outcomes. For example, disease-tailored CBT interventions have been shown to reduce depressive symptoms, pain severity and interference, in human immunodeficiency virus (HIV)-pain [21] and multiple sclerosis (MS)-pain [22]. Given the high comorbidity between chronic pain and depressive symptoms in IBD [4], CBT is a promising and appropriate psychotherapeutic approach to reduce pain-related distress and disability and to facilitate self-management.

**Fig. 1:**
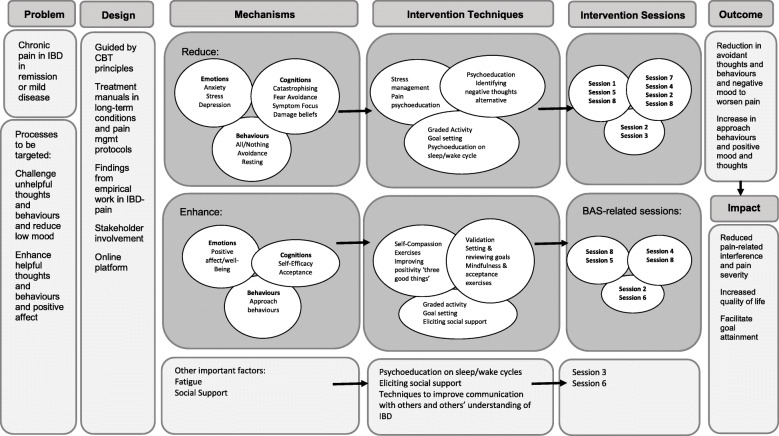
Logic model for IBD-pain self-management intervention: intervention development guided by empirical work on IBD-pain [4, 24, 25], stakeholder input and previous cognitive behavioural protocols for long-term conditions and chronic pain. Psychosocial factors associated with IBD-pain (mechanisms) to be targeted using cognitive behavioural techniques and mapped onto sessions to be included in the intervention (Table 1). This includes reducing psychosocial risk factors and enhancing protective psychosocial factors, as well as targeting other associated modifiable factors identified from empirical work on IBD-pain (fatigue, perceived social support) [4, 24, 25]. This is hypothesised to have an impact on reducing pain-related impact and severity and improve QoL

To our knowledge, no online CBT intervention has been tested and evaluated in the context of chronic IBD pain. The Medical Research Council (MRC) framework for developing complex health interventions [23] was used to guide the development of an online self-management intervention for IBD-pain. Intervention components were mapped onto key psychosocial processes associated with pain [4, 24] and incorporated input from patients and healthcare professionals to ensure that the intervention was relevant and tailored to people with IBD. A logic model, presented in Fig. 1, provides a summary of the psychosocial factors associated with IBD-pain (as evident from empirical work [4, 24, 25]) to be targeted through cognitive behavioural intervention techniques, with the aim of reducing pain-related disability and severity and improving QoL.

The aim of this study was to assess the feasibility and acceptability of an online facilitator-supported CBT-based self-management intervention, tailored for IBD-pain and delivered over 9 weeks. Prior to understanding feasibility of a future randomised controlled trial (e.g. recruitment and randomisation), we sought to understand feasibility of the intervention itself with a view to optimisation and further development if needed [26]. Specifically, our objectives were:

i) Explore adherence through completion rates and time spent on intervention sessions.
ii) Explore feasibility for the intervention facilitator through website usage and conducting a follow-up interview.
iii) Collect quantitative and qualitative measures of acceptability.
iv) Examine preliminary estimates of efficacy on pain-related interference and severity, QoL and psychosocial measures including emotions, cognitions and behaviours.
v) Explore response, recruitment and retention rates.

## Materials and methods

### Design

This was a single-arm pre-post feasibility study with a nested qualitative component. Full ethics approval was received from King’s College London KCL REC Committee, KCL ethics ID: RESCM-18/19-8806.

### Participants

Participants were recruited online via an IBD charity website (Crohn’s and Colitis UK—CCUK) and had previously completed an online survey about IBD-pain and consented to future research contact, or responded to an online advertisement. Eligibility and pre-screening criteria were as follows:

Inclusion criteria:

- Definitive diagnosis of inflammatory bowel disease received by a gastroenterologist
- Diagnosed with IBD for at least six months
- Aged 16 years of age or over
- Sufficient command of written and spoken English
- Chronic or intermittent pain experienced for at least 3 months or more

Exclusion criteria:

- An inability to provide informed consent
- Currently recruited into a pharmacological intervention/clinical trial
- Have recently undergone a course of CBT within the last six months
- Currently actively receiving psychotherapy or active psychological treatment.

Pre-screening criteria:

- Scoring of 4 or more out of 10 on the Brief Pain Inventory [27] (BPI) pain-interference scale, representing at least moderate pain-interference.
- No identified “red flags” indicating acute severe disease or pain likely to be attributed to other disease-related causes (see Supplementary Table 1). This was generated by a panel of expert IBD clinicians.

### Recruitment

Participants who had taken part in a previous IBD-pain questionnaire study and consented to future research were contacted by email and sent a Participant Information Sheet. Potentially interested participants were sent pre-screening and eligibility questions. If participants fulfilled these criteria, they were emailed with a consent form and were given the opportunity to ask any further questions. Recruitment took place over a 4-month period (February to May 2019). One hundred and forty-five individuals were contacted by email. Once all participants had been contacted from the cross-sectional study, recruitment was also advertised via a CCUK website page advertisement, to which two individuals responded expressing interest and were recruited into the study. All participants recruited into the study provided written informed consent.

### Procedure

Once participants provided informed consent to participate, they were emailed a link to the baseline questionnaire to complete online and provided with a unique study ID to ensure anonymity. Questionnaire items included sociodemographic and clinical data (IBD diagnosis, disease duration, IBD medication, pain medication), pain and psychological measures. Participants were sent a stool sample kit for faecal calprotectin via post and asked to return their stool sample to the laboratory. Remission status was defined by a faecal calprotectin score of < 250 ug/g. Once the baseline questionnaire was completed, participants were provided with a link to access the intervention. LS provided the participants with the opportunity to ask any questions before enrolling them onto the website. At week 9, participants were emailed a link to complete the post-intervention questionnaire and were asked if they were interested in participating in an interview to provide feedback over telephone/Skype or face to face. Follow-up feedback interviews lasted approximately 30 minutes per participant and were semi-structured using a topic guide (Supplementary Table 2) and probing questions and were carried out by a researcher (LS). An in-depth interview was also conducted with the intervention facilitator to further assess feasibility.

### Intervention

The intervention was developed by a team at King’s College London and guided by extensive stakeholder input, including people with IBD and IBD clinicians and other CBT-based protocols in long-term conditions [16, 19, 28]. It comprised of 9 sessions (Table 1) to be completed one session per week, with session 4a and 4b to be completed in week 4. Sessions were based on a CBT framework and included tasks for participants to complete in their own time, such as reviewing set goals or practising skills learnt within the sessions. Sessions and tasks were interactive, allowing for participants to select items or write text relevant to them and included audio and video clips. Participants were invited to complete their own “vicious cycle”; a visual diagram of the individual’s personal model of their thoughts, emotions and behaviours associated with their IBD-pain. Character illustrations and patient vignettes were also provided throughout sessions to contextualise examples and provide stories of people with IBD. The intervention was delivered through an online platform called “BOOST”, developed for a larger RCT study on the management of pain, fatigue and urgency in IBD. Participants were instructed to complete core sessions (1–6 and 9) and pain-specific sessions (7 and 8) for the 9-week programme and not select symptom-specific sessions on fatigue and urgency (but had access).

**Table 1:** Intervention sessions, brief summary of content and tasks to complete

Session title	Session content	Session task
Session 1: Understanding your IBD symptoms	Factors contributing to pain, fatigue and urgency, looking at the vicious cycle, use of self-monitoring and setting programme aims.	Symptom diary
	**intervention facilitator phone call**	
Session 2: Balancing your activity, eating and exercise	The importance of activity and exercise and looking at the fear avoidance model, eating patterns and setting goals.	Working towards and reviewing goals for activity. Sleep diary
Session 3: Improving your sleep	The importance of sleep and looking at different sleeping patterns and habits. Techniques to improve your sleep. Setting goals.	Working towards and reviewing goals for sleep
Session 4a: Changing your thoughts: Part 1	The contribution of thoughts and the impact of these on pain. Identifying unhelpful thinking.	Keeping a thought record in the context of pain
Session 4b: Changing your thoughts: Part 2	Developing alternating thoughts in the context of pain.	Keeping a thought record and coming up with alternatives
Session 5: Managing stress and coping with emotions	The effects of stress and how to manage it, including mindfulness exercises. Looking at different emotions. Setting goals	Working towards and reviewing goals for managing stress and keeping a stress diary
Session 6: Making the most out of your social support and communication	Looking at different types of social support. Improving communication and disclosure. Setting goals	Working towards and reviewing goals for social support and communication
Session 7: Managing and understanding pain in IBD	Difference between acute and chronic pain, cause of IBD-pain, looking at the vicious cycle in the context of IBD-pain.	
Session 8: The role of acceptance and self-compassion in pain	How can acceptance help me? Looking at resilience, and self-compassion exercises.	
Session 9: Summary and maintaining improvement	Revisiting programme aims, preparing for the future, sustaining improvements and building on them.	

### Facilitator support

After completing session 1 in the programme, participants were invited to speak with the intervention facilitator over the telephone (for approximately 30 minutes) to review session 1, the participant’s personal cognitive behavioural model and their programme aims. Participants were also able to contact the intervention facilitator through in-site messaging on the website. The facilitator for this study was a mental health nurse, who received training in CBT from RMM prior to carrying out phone calls. To ensure treatment fidelity, the facilitator had regular supervision from the BOOST intervention team and used a checklist and prompt sheet to structure telephone sessions.

### Feasibility outcomes

Data collection for feasibility included measuring the proportion (percentage) of individuals eligible from the online cohort, participants consenting and agreeing to take part in the study and participants completing the post-intervention questionnaire (retention). Withdrawal and drop-out rates and compliance to and completion of treatment sessions and session tasks were also assessed. Session/task completion and duration were collected through a website data usage function.

### Acceptability

Acceptability was measured quantitatively and qualitatively through seven key areas assessing cognitive and emotional responses from participants, as proposed by a theoretical framework of acceptability [29]. This included affective attitude towards the intervention (positivity), how much effort was perceived by individuals, to what extent the intervention was effective, how helpful the intervention was perceived to be, to what extent participants understood the workings of the intervention, how confident participants felt to complete the session and tasks within the interventions and how costly partaking in the intervention was for participants (effort, time spent, resources etc.). These were measured by 7 items using a visual analogue scale (0–10) in a bespoke questionnaire of acceptability at post-intervention (Supplementary Table 3). Overall acceptability was indicated through the total score (0–70). The seven constructs were also embedded within the topic guide (Supplementary Table 3) utilised in semi-structured interviews for qualitative feedback on participant experiences of the intervention.

### Secondary outcome measures

#### Pain-related interference and pain severity

Pain-related interference and pain severity were measured using the Brief Pain Inventory (BPI) [27]. Pain-related interference is measured in seven domains including the impact of pain on relationships, work, sleep and exercise ranging between 0 (does not interfere) to 10 (completely interferes). Pain severity is assessed by 4 items, including pain at the worst and least in the previous 24 h, pain severity on average and pain “right now” ranging from 0 (no pain) to 10 (pain as bad as you can imagine). BPI also asks for ratings on the extent of relief pain medications have provided in the prior 24 h.

#### Quality of life

The United Kingdom (UK) version of the Inflammatory Bowel Disease Questionnaire (UKIBDQ) [30] was used to measure health-related QoL. This is a UK version of the McMaster IBDQ [31] and is a 32-item questionnaire assessing various aspects of health-related QoL. Questions assess aspects of QoL including bowel, emotional, physical and social functioning. Summary scores range from 30–120, with lower scores indicating poorer health-related QoL.

#### Disease activity

Disease activity was measured through the IBD-control questionnaire [32]. This is a 13-item measure that assesses disease control from a patient perspective, including items on change in bowel systems or IBD treatment, impact and overall rating of control in the past 2 weeks.

#### Depression

Patient Health Questionnaire-9 (PHQ-9) was used to measure depression [33]. The PHQ-9 assesses symptoms within the prior 2 weeks using a 4-point Likert scale ranging from “never” to “nearly every day”. Greater scores represent greater number of depressive symptoms, with a scoring of 0–4 representing minimum, 5–9 mild, 10–14 moderate, 15–19 moderate to severe and > 20 representative of severe depressive symptoms.

#### Anxiety

The Visceral Sensitivity Index (VSI) [34] was used to measure gastrointestinal-specific anxiety. This is a 15-item scale with items assessing affective, cognitive and behavioural aspects of anticipatory occurrence of symptoms, rated on a 1–6 scale (strongly disagree to strongly agree). Items include “I constantly think about what is happening inside my belly”, with greater scores indicating greater gastrointestinal-specific anxiety.

#### Pain catastrophising

Pain Catastrophising Scale [35] was used to assess thoughts that involve rumination, helplessness or magnification which exaggerate the threat of pain sensations. The scale is comprised of 13 items with a Likert range of 0–4 (Not at all to All the time) with greater scores indicating greater extent of catastrophising thoughts.

#### Cognitive and behavioural responses to pain; fear avoidance, avoidance resting behaviour and all or nothing behaviour

One cognitive and two behavioural sub-scales from the Cognitive Behavioural Responses to Symptoms Questionnaire (CBRQ) [36] were used to measure Fear Avoidance, Avoidance Resting Behaviour and All or Nothing Behaviour. Fear Avoidance sub-scale responses range from “strongly disagree” to “strongly agree” and behavioural sub-scales rated from “never” to “all the time”.

#### Pain self-efficacy

Pain Self-Efficacy questionnaire [37] assesses the extent to which an individual’s believe they can carry out actions and tasks, despite their pain. Scores are rated from 0 to 60 with higher scores indicating greater self-efficacy.

#### Resilience

Connor-Davidson Resilience Scale (CD-RISC)-10 was used to measure self-perceived resilience [38]. The overall CD-RISC 10 score ranges from 0 to 40, where higher scores represent greater resilience.

#### Statistical analysis

A sample size calculation was not required a priori for this single-arm feasibility study. Consulting guidelines on sample size for pilot studies [39] and a similar intervention in IBD [19], a sample size aim of 20 participants per arm was deemed adequate to assess for feasibility and acceptability, and allow for 70% retention rate. Descriptive data were presented as means and standard deviations (continuous variables) and percentages (categorical variables) at baseline and post-intervention (week 9). Paired sample *t*-tests were used to compare participants at baseline who completed the post-intervention questionnaire or were lost at post-intervention data collection. Statistical analyses were carried out using SPSS (Version 25).

#### Qualitative analysis

Inductive thematic analysis was used to analyse interviews [40, 41]. The audio recordings were transcribed by a third-party transcriber and the lead author read and re-read all transcripts. Pen and paper line by line coding was conducted to generate codes and initial themes, and the lead author consulted with all authors to discuss and decide on final themes.

## Results

### Sample characteristics

Twenty participants were recruited and consented to the study. Sociodemographic and clinical characteristics of the study sample are presented in Table 2. The mean age of participants was 38.4 years (range 22–58 years) and the majority were female (80%), of White British origin (95%) and had a diagnosis of CD (80%). No recruited participants had a diagnosis of IBD-unclassified. Mean scores for pain, QoL, self-reported disease activity and psychological outcomes for the overall sample at baseline are presented in Supplementary Table 4. Characteristics of the baseline and post-intervention (analysis) group were similar. Of the 20 participants, 15 (75%) returned a stool sample, of whom 12 (60%) were in clinical remission. In the 16 participants who completed the post-intervention questionnaire, 13 participants returned a stool sample for faecal calprotectin, of whom 10 (76.92%) were in clinical remission.

**Table 2:** Baseline sociodemographic and clinical characteristics of 20 consenting participants

Characteristic	Recruited participants (***n*** = 20)
**Sociodemographic**	
Age, mean (SD), yr	38.40 (9.87)
Female	16 (80)
English/Welsh/Scottish/N Irish/British	19 (95)
University degree or higher	13 (65)
***Employment status***	
Employed full-time	10 (50)
Employed part-time	2
Full- or part-time education	2
Full-time domestic	1 (5)
Retired	1 (5)
Unemployed	4 (20)
***Relationship status***	
Married/civil partnership	9 (45)
Living with partner	5 (25)
Divorced	1 (5)
Single	5 (25)
**Clinical**	
Diagnosis (CD/UC/IBD-U)	16 (80)/4 (20)/0 (0)
Disease duration mean (SD) (yrs)	13.20 (10.46)
Faecal calprotectin mean (SD) ug/g	175.69 (205.69)
***IBD medication***	
5-ASA	6 (30)
Thiopurines	4 (20)
Anti-TNF (infliximab/adalimumab)	3 (15)/4 (20)
Vedolizumab	1 (5)
Steroids (methotrexate/budesonide/prednisolone)	3 (15) /3 (15) /2 (10)
Ustekinumab	5 (25)
None	2 (10)
Previous surgery	12 (60)
***Surgeries***	
Resection	5 (25)
Colectomy	3 (15)
Stoma	2 (10)
***Smoking history***	
Current smoker	3 (15)
Previous smoker	5 (25)
Non-smoker	12 (60)
***Pain locations***	
Abdomen	18 (85)
Joints	14 (70)
Back	13 (65)
Head	8 (40)
**Pain medications**	
Paracetamol	10 (50)
Co-codamol	4 (20)
Opioids	10 (50)
Pregabalin	1 (5)
NSAIDs	3 (15)
Tricyclic antidepressants	3 (15)
Antispasmodics	1 (5)
Omeprazole	1 (5)
Benzodiazepine	1 (5)
Other (anti-diarrhoeal, anti-bile acid)	0 (0)

*NB* count displayed as % unless otherwise specified

### Feasibility: response and recruitment rates

**Fig. 2:**
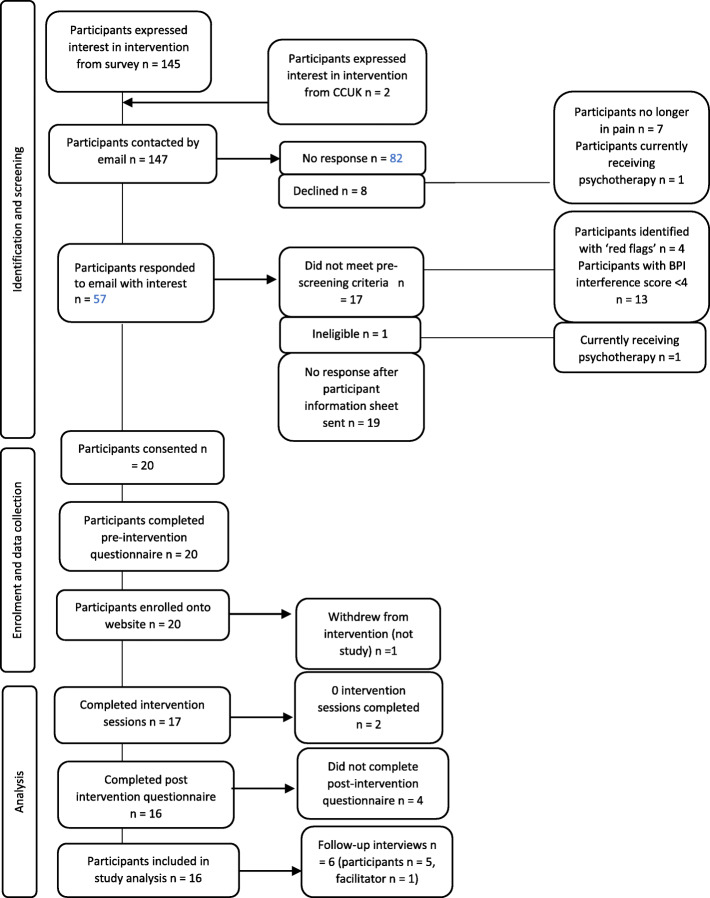
Study flow diagram of participants approached, consented and recruited into study

Figure 2 presents the participants’ flow in identification, screening, enrolment, data collection and analysis stages. Of the 145 individuals approached about the study, 55 individuals responded (37.9% response rate). In some cases, participants would express initial interest to partake in the study and then not make further contact with the research team. The recruitment rate may be explained by complications within email methods, as emails may have not reached potentially interested participants (e.g. getting lost to junk/spam folders as was initially reported by some participants). Once expressing interest and reaching the pre-screening stage, a large proportion of individuals either presented “red flags” (clinical symptoms indicating active disease or complications) or scored less than 4 on the pain-related interference questionnaire, resulting in a recruitment rate of 36.4% from those who responded to the email invitation.

Twenty individuals (inclusive of two individuals expressing interest from CCUK advert) with IBD met pre-screening and eligibility criteria and provided informed consent (35.1% recruitment rate from those who responded to the email or contacted the research team from study advertisement).

### Feasibility: retention rates

Of the 20 participants who consented to the study, 20 (100%) completed the baseline questionnaire and were enrolled on the website. One participant withdrew from the intervention (but not the overall study, agreeing to complete the post-intervention questionnaire at week 9). From the 19 remaining individuals, 17 completed intervention sessions (85%). Of the 9 sessions, 2 (10.5%) completed 0 sessions, 2 (10.5%) completed 1–2 sessions, 4 (21.1%) completed 3–5 sessions, 9 (47.4%) completed 5–7 sessions and 2 (5.3%) completed all 9 sessions. 16 participants from the 20 recruited and consented to the study completed the post-intervention questionnaire at week 9 (80%). Analyses found that participants who did not complete follow-up questionnaire data (*n* = 4) did not significantly differ to completers (*n* = 16) in baseline pain severity (*p* = .511) or pain-related interference (p = .164), age (*p* = .259), employment (*p* = .113) or educational status (*p* = .876). Mean disease duration was significantly lower in post-intervention questionnaire completers (mean = 10.9 years) compared with non-completers (mean = 22.5 years, *p* = .043). QoL was higher and self-reported disease activity and avoidance resting behaviour was lower in post-intervention questionnaire completers (*p* < .05). All remaining psychological factors did not significantly differ between post-intervention questionnaire completers and non-completers at baseline.

### Feasibility: participant usage and facilitator usage

On average, a mean number of 3 messages were sent from participants (*n* = 19) to the facilitator (min 0, max 8) and the mean average of 3 messages were sent from the facilitator to the patient. Participants spent a mean time of 19.84 minutes on each session (median 14 minutes, range 1–100 minutes). Out of a scoring of 5, mean scores across sessions on helpfulness = 4.1, relevance = 4, easy to navigate = 4.4 and motivation to use strategies covered = 3.6. 18 out of 20 participants responded to the facilitator in-site message and carried out a facilitator phone call at week 1, while 2 participants did not respond to any facilitator messages nor complete any intervention sessions after consenting to the study.

### Feasibility: qualitative feedback from intervention facilitator

A follow-up interview with the intervention facilitator further supported that the intervention was feasible. Feedback was divided into themes around i) phone call and website usage ii) preparations and training and iii) provision of support.

i) Telephone and website usage

The facilitator said that telephone sessions generally kept to time and were easy to schedule with the patients using the in-site messaging and personalised calendar.

However, the facilitator shared that they felt another phone call halfway through, or at the end of the intervention, would have been beneficial, “to say ‘well done for completing it or let’s review some of your reflections’”.

ii) Preparations and training

Despite little knowledge of CBT or IBD before the study, the facilitator said that adequate training and education in both helped them feel prepared. Resources provided to the facilitator such as prompt sheets and the patient’s online populated vicious cycle were useful to assess and have throughout the telephone sessions with patients:

“The script kept me on a structure and a focus to make sure I was covering what I needed to”.

iii) Provision of support

While the majority of participants messaged infrequently after they had had their telephone session, a small number messaged throughout, to which the facilitator felt they had provided adequate support and feedback:

“One patient messaged me frequently as they felt at times unwell and anxious that they wouldn’t meet the timescale to complete the intervention. I offered them reassurance and understanding”.

### Acceptability: quantitative findings

The mean acceptability scores for the seven constructs, and the total overall score rating for acceptability (0–100) are presented in Table 3.

**Table 3:** Mean scores for seven acceptability constructs measured in post-intervention questionnaire

Acceptability construct (0–10) (***n*** = 16)	Mean (SD)	Min–max
Positivity	6.25 (3.19)	0**–**10
Effortful	6.31 (1.82)	2**–**9
Effectiveness	5.38 (3.30)	0**–**10
Helpful	5.12 (3.24)	0**–**10
Understand workings of intervention	8.56 (1.97)	5**–**10
Confidence to complete	8.19 (2.01)	4**–**10
Costly (time, resources)	3.50 (2.99)	0**–**8
Overall acceptability	43.31 (11.31)	20**–**62

### Qualitative feedback: participants

Acceptability of the intervention was also assessed through semi-structured interviews. Five participants responded to an invitation for an interview to feedback on their experiences of the intervention. Three themes were identified. These were “Facilitator and individual input”, “Thoughts and reflections” and “Content and format”. These were broken down into sub-themes and are described below, illustrated by verbatim quotes and demographic information (participant ID, gender, age, diagnosis, disease duration in years) of interviewees in brackets.

1. Facilitator and individual input

i) Knowing someone was there

Participants were positive about the facilitator role, agreeing that even though they did not feel the need to be in frequent contact, having a facilitator gave them a source of support and a personal element to the programme:

“I felt I knew she was there if I needed her but at the same time, I was able to take things on board and didn’t feel the need to go back [to her]” (P18, Female, 43, CD, 4).

“It was nice to touch base and know there is a human being behind what’s happening” (P4, Female, 24, UC, 3).

However, one participant felt that more contact would have been beneficial:

“I feel I would have liked more contact with her on maybe a fortnightly basis, in the form of her dropping a message to see how I was getting on” (P5, Female, 24, CD, 5).

ii) Self-management and control

While there was appreciation for facilitator support, there was a sense of empowerment that came out of individual work and input into the programme. This included developing a greater sense of control and autonomy:

“That’s how I viewed the study, not that it was going to take my pain away but that it is the mechanism as to how you manage it and put you back in control” (P18, Female, 43, CD, 4).

“Going through these sessions its actually making me realise, as people we are really powerful and we can take a lot on for ourselves” (P5, Female, 24, UC, 5).

*…* and the ability to change things for oneself by practising the techniques from the sessions:

“For me it was the thoughts session which I thought I can change myself quite quickly” (P10, Female, 23, CD, 12).

2. Thoughts and reflection

i) Usefulness of thoughts session

The sessions on thoughts appeared to be the most helpful for participants and the skills that they would most likely take forward and continue to practise in their daily lives:

“For me it was being able to identify the thoughts and rationalise them in a much better way” (P18, Female, 43, CD, 4).

“[I’m] definitely keeping up with the exercises and thought processes, the coming up with alternatives, I sort of do that in my head now” (P10, Female, 24, CD, 12).

For one participant, this skill helped “set them on the right path” and as previously they had been “stuck in a rut” (P4, Female, 24, UC, 3).

ii) Understanding and responding to pain

The exercises and content within the intervention enabled participants to have a better understanding of their pain, which had a positive impact on the way they responded to their pain. In particular, the use of the vicious cycle exercise was helpful in allowing individuals to “break things down” (P18, Female, 43, CD, 4) and rationalise their experience.

“The aspects on identifying my stressors and my vicious cycle was interesting as it helps me understand the thought processes going on when I am in pain” (P5, Female, 24, UC, 5).

“It’s helping with my confidence in that way to think okay that’s just normalised pain as opposed to specific pain that’s sharper” (P4, Female, 24, UC, 3).

3. Content and Format

i) Content and tone

Participants provided feedback on the content and tone of the intervention; with some of it being a “little repetitive” *(*P4, Female, 24, UC, 3) and more than one feeling that the tone was overly patronising. One participant commented that some of the content was over-simplified such as consistent pattern of eating and regular exercise, as they highlighted the complexity of diet in IBD and that exercise was not easy to achieve, given their symptoms and health. This was shared by others who commented on the brief content covered on diet, within the exercise and activity session.

One participant noted that there were not enough practical tips offered in the intervention to help people with their pain:

“There are other natural things like hot water bottles and certain stretches and exercises that are good for pain, actual practical self-management tips for pain which are non-medicinal. It would be nice to have something similar to this, for times of acute pain” (P4, Female, 24, UC, 3).

However, other participants found the varied layout and format of content was a good way of presenting lots of information, and the use of vignettes helped with putting session content into the context of IBD.

ii) Length and workload

Some sessions, such as the ‘Managing Stress and Coping with Emotions’ session was felt too long by participants and that the weekly completion of sessions and tasks was too demanding for some who were impacted by multiple symptoms of their IBD.

*“*I found the [tasks] difficult to complete on a weekly basis simply due to life getting in the way and extreme fatigue and pain that my current flare has been causing” (P5, Female, 24, UC, 5).

One participant also commented that the repetitive goal setting was overloading for them, while others liked this repetitive structure of goal setting associated with tasks.

iii) Online platform

The advantages of using an online platform were commented on, as this facilitated self-management and overcame barriers of face-to-face therapy.

*“*It was nice to be able to do [it] in my own environment and as and when I had the time”. (P4, Female, 24, UC, 3).

“Seeing the questions and having the scenarios, I could take it on board more” (P18, Female, 43, CD, 4).

However, one participant commented on the limitations of the use of an interactive website as the options “narrow[ed] down’ individuals” issues into “fixed categories” (P2, Female, 48, CD, 25) and that the structure of the programme prevented them from accessing later more relevant sessions at an earlier stage.

Several of the participants shared that the online intervention was timely and well received. For some, this was because they felt that although their medications were controlled, they did not feel in control of their IBD symptoms, while for others, they had been on a waitlist to see a psychologist and this platform allowed them to overcome this inaccessibility and regain a sense of self-management.

#### Secondary outcomes—changes to pain, QoL and disease activity

Table 4 presents mean scores and standard deviations for secondary outcomes at baseline and post-intervention for the 16 participants with both measures. Small decreasing effects were seen in pain-related interference and pain severity, whereas a greater difference was evident in improvement of QoL at post-intervention (Cohen’s *d* = 0.72). Disease activity measured through the IBD-control also showed improvement at post-intervention.

**Table 4:** Mean scores at baseline and post-intervention for pain outcomes

Mean (SD)	Baseline	Post-intervention	Paired mean differences	95% CI
	***n*** = 16	***n*** = 16		
Pain interference (0–10)	5.90 (1.69)	5.03 (1.81)	0.87	− 0.31, 2.06
Pain severity (0–10)	4.92 (0.92)	4.45 (1.83)	0.47	− 0.36, 1.26
Quality of life (30–120)	74.94 (10.38)	82.94 (11.94)	− 8.00	− 13.63, − 2.37
Depression (0–27)	14.19 (4.90)	10.69 (6.86)	3.50	0.22, 6.78
Anxiety (0–75)	58.56 (15.31)	52.75 (16.99)	5.81	0.17, 11.45
Pain catastrophising (0–52)	22.73 (9.18)	16.27 (10.42)	6.46	0.95, 11.98
Fear avoidance (0–24)	13.13 (4.21)	12.06 (5.79)	1.07	− 0.79, 2.13
Pain self-efficacy (0–60)	27.25 (6.27)	30.88 (9.62)	− 3.63	− 8.41, 1.16
Resilience (0–40)	25.38 (5.06)	25.00 (5.59)	0.38	− 1.99, 2.74
Avoidance resting (0–32)	14.20 (4.80)	11.13 (4.79)	3.07	0.11, 6.02
All or nothing (0–16)	12.93 (4.03)	12.67 (5.27)	0.26	− 2.15, 2.68
IBD control (0–100)	67.31 (15.50)	75.94 (17.99)	− 8.63	− 18.06, 0.81

*QoL* psychological factors and disease activity, paired mean differences and 95% confidence intervals (CI) for paired sample *t*-test between baseline and post-intervention scores

#### Secondary outcomes—changes psychological outcomes

Depression and anxiety both decreased with medium effect sizes. Pain catastrophising scores decreased on average by 6.45 at post-intervention. Self-efficacy, resilience or fear avoidance demonstrated minimal changes between the two time points; however, avoidance resting behaviour showed reductions with a medium effect size at post-intervention (Cohen’s *d* = 0.64).

## Discussion

The aim of this study was to test the feasibility and acceptability of a therapist-supported online CBT-based self-management intervention for chronic pain in IBD. Content in the programme was well received by individuals; participants were positive about the online format, the thoughts session, “breaking things down” and empowering individuals to feel more in control of their pain. This supports the theory underpinning the intervention as described by the logic model (Fig. 1), whereby cognitive behavioural techniques aim to build self-efficacy [42] and skills around thought monitoring to reduce pain-related impact and improve QoL.

Several participants felt that some of the content was patronising and over-simplified, particularly given the complexity of certain aspects of IBD, such as diet and difficulties of engaging in exercise. It was concluded by the research team that given the heterogeneity in IBD diet research, covering diet in depth was not within the remit and aims of this intervention. However, further work should aim to understand how information and development of skills regarding diet can be implemented into a self-management intervention for pain in IBD.

As evident from qualitative feedback, the effect of having a facilitator included in the programme for support and reflection was positive, despite some participants not feeling the need to regularly message the facilitator on a daily or weekly basis. This is important given the findings in previous online CBT interventions in IBD, which found high drop-out rates in the absence of any therapist support [17]. Evidently, some level of therapeutic engagement is important for sustained motivation and support when individuals take part in a psychological intervention in this population. An average of 3 messages were received by the facilitator, exemplifying the feasibility of the use of an online messaging platform to converse with patients in future online interventions in IBD.

Response rates following invitation to the study were not as high as anticipated (37.9%), which may have been a result of email contact only rather than face-to-face recruitment. As just over a third of invited participants were eligible for the study, it may have been that the screening criteria threshold for pain-related interference was too high; however, a moderate pain intensity score of 4 or more has been used as a threshold for chronic pain interventions studies in other long term conditions [22] and ensured that those with the capacity to benefit were included.

At least 70% of participants completed three or more treatment sessions and over half of participants completed at least 5 out of 9 sessions. These findings are similar to previous CBT intervention studies in IBD [17] (where 48.8% of participants completed more than 4 sessions) and in HIV-chronic pain [21] and therefore deemed feasibility of the intervention. Two participants did not complete any sessions and one dropped out of the intervention after session 1, as they felt the content was not helpful for them. Unfortunately, those who completed no sessions did not reply to the research team after multiple contacts, including invitation to be interviewed. Reasons for noncompletion may have been general disengagement with the programme, business of daily life or, due to the relapsing-remitting nature of IBD, coping with a flare or general challenges of living with IBD. Indeed, one participant in qualitative feedback shared that weekly completion of sessions and tasks was too demanding given their illness and current levels of pain and fatigue while undertaking the intervention.

Issues around adherence to digitally-delivered complex health interventions have been recognised in the literature. Ryan et al. [43] highlight the need for an interdisciplinary approach between individual, environmental and technological factors to promote better engagement to web-based interventions. Moreover, understanding factors associated with both adherence (frequency and duration of engagement) and attrition (halting engagement altogether) is needed so we can better support patients when developing future interventions [44].

In a qualitative meta-synthesis of user experience from computerised therapy for anxiety and depression, personalisation and sensitivity of content were highlighted as important areas in facilitating user engagement [45]. While strengths of computerised CBT lie in aspects such as privacy, independence and self-mastery, it is important that interventions still strive to foster connectivity and collaboration, as these are vital parts of the therapeutic process in CBT [45]. The development of the BOOST intervention tested in this feasibility study involved extensive stakeholder input. Content was tailored specifically for people with IBD. However, it may have been that greater personalisation and collaboration through more regular (e.g. weekly) facilitator messaging required for better engagement. Future patient and public involvement to understand individual and environmental factors that may act as barriers or facilitators to intervention engagement will help refine intervention design and optimise outcomes. This has since been carried out by the BOOST intervention development team with extensive stakeholder involvement and has helped further inform intervention development.

Those completing the post-intervention questionnaire had greater baseline QoL and lower self-reported disease activity, suggesting that individuals with poorer health and QoL either require greater support throughout the programme or medical management to be optimised more thoroughly before entering the programme. Unlike previous RCTs, this study did not find effects of baseline psychological distress on retention rates [46]. Participants who did not complete the post-intervention questionnaire had been diagnosed with IBD for a significantly greater time period. It may be that individuals who have had a longer diagnosis may be less likely to engage in such interventions, possibly because they have tried psychotherapy previously, have had a greater length of time to adjust to their IBD or have greater understanding of their symptoms and aetiology compared with those more recently diagnosed. Participants who did not complete the intervention did not significantly differ from completers on pain, sociodemographic or any other clinic outcomes.

The study was not adequately powered to detect efficacy. However, results tentatively suggest that the intervention could have positive effects in reducing emotional distress and pain-related thoughts and behaviours. Scores in resilience showed no change during the programme. While one could argue that resilience may be more a trait than a state factor, a systematic review of “resilience training programmes” in individuals with chronic conditions found a modest but consistent benefit on several mental health outcomes, suggesting that resilience can be modified by an intervention [47]. Leppin and colleagues contend that resilience is a “modifiable construct and not an inherent, immovable trait of individuals” [47] (pg. 2). Furthermore, a large proportion of these reviewed studies used the CD-RISC measure, as was used in the current study. While pain self-efficacy showed small to moderate improvements, an overall smaller effect on positive psychosocial outcomes may have been explained by a lesser focus on positive psychological techniques, which was included in one out of the nine sessions, compared with the majority of sessions aiming to modify negative or unhelpful psychosocial processes. Alternatively, it may be that negative psychological processes are more amenable to change through intervention compared with positive, or that “resilience states” are context dependent. Future research should endeavour to examine whether an intervention with greater emphasis on positive psychological intervention techniques results in improvement in resilience, self-efficacy and other positive psychological factors in IBD (e.g. optimism, self-regulation, perception of social support).

### Strengths and limitations

A key strength of this study was the use of the MRC framework for complex health interventions [23] to guide development of this intervention, which facilitated the acceptability and feasibility of this intervention. This included extensive stakeholder involvement to ensure that content was relevant and tailored to individuals with IBD and chronic pain, and that session and task length was feasible given the demands of living with a chronic condition. Key limitations include the lack of both online and face-to-face recruitment methods to optimise recruitment rates, and a control group to determine feasibility of randomisation, control group effects and initial estimates of efficacy in comparison with an active treatment arm, treatment as usual or waitlist control. Clinical data on recruited participants was only collected at baseline; however, collection of this data at post-intervention may have revealed interesting findings (e.g. change in pain medication). Future pain interventions in IBD should aim to collect this data at pre- and post-time intervals.

A non-validated theory-based measure was used to quantitatively assess acceptability, and further research is required to validate this quantitative measure of acceptability using Sekhon’s theoretical framework [29]. Finally, nested qualitative interviews were carried out by LS who was involved in the intervention development, and therefore social desirability bias may have been pertinent during interviews and limited the information and views shared by participants. Participants who completed few or no treatment sessions were not interviewed, therefore issues regarding feasibility and acceptability for these participants were not collected. Additionally, given that the majority of participants were white and female, and there is underrepresentation here, and more broadly of black, ethnic and older groups in computerised therapy interventions [45], further work is needed with a more diverse sample to understand generalisability of results.

## Conclusion

This study has demonstrated the feasibility and acceptability of an online CBT-based self-management intervention for IBD-related chronic pain. The content, online format and facilitator support were well received by participants and preliminary estimates of efficacy demonstrated improvements in QoL and reductions in negative affect and pain-related thoughts and behaviours. Recommendations for future research include further collection of qualitative data from people with IBD to understand barriers and facilitators to engagement and identify key effective components of the intervention to refine content. In addition, feasibility testing of trial procedures including willingness to be randomised and subsequently a large-scale RCT is recommended to confirm efficacy of the intervention, using a combination of face-to-face and online recruitment methods to optimise uptake.

## Supplementary Information

**Additional file 1: Supplementary Table 1**. “Red Flags” checklist to identify pain likely attributed to medical causes/IBD flare. **Supplementary Table 2**. Topic guide for nested qualitative interviews post-intervention. **Supplementary Table 3**. Questionnaire items representing seven components of acceptability based on theoretical framework by Sekhon et al. [29]. **Supplementary Table 4**. Baseline pain, quality of life, psychological factors and disease activity scores for total sample (*n* = 20)

## Data Availability

The datasets generated during and/or analysed during the current study are not publicly available (individual privacy of the participants could be compromised) but are available from the corresponding author on reasonable request.
