# Supplementary material for: Pain management in inflammatory bowel disease: feasibility of an online therapist-supported CBT-based self-management intervention
Source: Pilot Feasibility Stud. 2021 Apr 16;7:95. doi: 10.1186/s40814-021-00829-9 (PMC8050888; doi:10.1186/s40814-021-00829-9)
Supplement: Supplementary file 1 — Additional file 1: Supplementary Table 1. “Red Flags” checklist to identify pain likely attributed to medical causes/IBD flare. Supplementary Table 2. Topic guide for nested qualitative interviews post-intervention. Supplementary Table 3. Questionnaire items representing seven components of acceptability based on theoretical framework by Sekhon et al. [29]. Supplementary Table 4. Baseline pain, quality of life, psychological factors and disease activity scores for total sample (n = 20) [file 40814_2021_829_MOESM1_ESM.docx]

**Supplementary Materials – Feasibility of Online CBT intervention for pain in Inflammatory b**

**Bowel Disease**

|  |  |  |
| --- | --- | --- |
| 1. New (over the last 2 weeks) severe pain in your stomach (upper abdomen) which may or may not be much worse after eating?  If yes:  1a: Has this been present for 3 months or more?  1b: Have you spoken to a doctor or nurse about this pain?  1c: Have you had any investigations for this pain?  1d: Has any cause been diagnosed for this pain? If so, what?........................................................................................ | Yes  Yes  Yes  Yes  Yes | No  No  No  No  No |
| 2. Regular or persistent vomiting over the past 2 weeks | Yes | No |
| 3. Unintentional rapid weight loss of 5kg (10 pounds) or more (over 2-3 months) without trying to lose weight | Yes | No |
| 4. New (over the past 2 weeks) inability to eat anything except a very soft diet because solid food causes pain | Yes | No |
| 5. Sudden new onset of constipation plus inability to pass gas (flatus) and a very distended abdomen over the past 2 weeks  If yes:  5a: Has this been present for 3 months or more?  15b: Have you spoken to a doctor or nurse about this pain?  5c: Have you had any investigations for this pain?  5d: Has any cause been diagnosed for this constipation? If so, what?........................................................................................ | Yes  Yes  Yes  Yes  Yes | No  No  No  No  No |
| 6. New (over the past 2 weeks) symptoms of passing dark black (treacle-like) stools which has NOT been investigated | Yes | No |
| 7. Fevers (high temperature, except for an obvious reason, such as the ‘flu’) over the past 2 weeks which has NOT been investigated | Yes | No |
| 8. New rectal bleeding (more than a few drops) over the past 2 weeks which has NOT been investigated | Yes | No |

**Supplementary Table 1. “Red Flags” checklist to identify pain likely attributed to medical causes/IBD flare**

**Supplementary Table 2. Topic guide for nested qualitative interviews post-intervention**

| **Questions** | **Prompts** |
| --- | --- |
| Tell me about your IBD | What type of IBD do you have?  How long have you had IBD for?  What are the main symptoms of your IBD?  Which medications are you taking for your IBD? |
| Tell me about your experience of the intervention | How has the intervention been for you?  Can you tell me more about that?  Can you tell me what you liked about the intervention?  Can you tell me what you didn’t like about the intervention? |
| Tell me about your experiences of and views on the sessions and homework tasks | Tell me about your experiences of carrying out the activities in the sessions  Tell me about your experiences of carrying out the homework tasks? |
| Tell me about your experiences of and views on the support from the facilitator | What did you find helpful from your discussions with the facilitator?  What would you have liked to gain more of from your discussions with the facilitator? |
| How useful was the intervention for you? | Which parts of the intervention were most helpful and why?  Which parts of the intervention were least helpful and how can these be improved? |
| How do you think the intervention could be improved? | How do you think the format could be changed (changes in number of sessions, modality of delivery, manual format, language)?  How could the delivery of the intervention be improved?  What information was missing from the manual?  What additional information could be included?  Are there any parts of the intervention that you would like to continue using?  Do you have ideas of other ways in which pain in IBD could be managed that were not included in this intervention? |
| Final comments | Is there anything else you would like to add or talk about? |

**Supplementary Table 3. Questionnaire items representing seven components of acceptability based on theoretical framework by Sekhon et al. (29)**

| **Item** | **Acceptability component** | **Question** |
| --- | --- | --- |
| 1 | *Affective Attitude* | How positive do you feel about the intervention? |
| 2 | *Burden* | How much of an effort was the intervention? |
| 3 | *Perceived Effectiveness* | To what extent did you perceive the intervention to be effective? |
| 4 | *Ethicality* | To what extent did you find the intervention a helpful approach for managing your pain? |
| 5 | *Intervention Coherence* | To what extent did you understand the workings of the intervention? |
| 6 | *Self-Efficacy* | How confident did you feel to participate in the sessions and tasks in the intervention? |
| 7 | *Opportunity Costs* | To what extent was the intervention costly for you? (i.e. time given up doing other things) |

**Supplementary Table 4.** Baseline pain, quality of life, psychological factors and disease activity scores for total sample (n = 20)

| **Measure** | **Mean (SD)** |
| --- | --- |
| **Pain interference (0-10)**  **Pain severity (0-10)**  **Quality of life (30-120)**  **Depression (0-27)**  **Anxiety (0-75)**  **Pain catastrophising (0-52)**  **Fear avoidance (0-24)**  **Pain self-efficacy (0-60)**  **Resilience (0-40)**  **Avoidance resting (0-32)**  **All or nothing (0-16)**  **IBD control (0-100)** | 6.19 (1.80)  5.01 (1.19)  71.90 (12.10)  14.45 (5.88)  60.01 (16.54)  22.10 (12.71)  13.20 (4.09)  25.40 (9.023)  25.60 (6.39)  15.15 (5.46)  12.80 (4.75)  62.25 (17.53) |
